# Yield-strength prediction of flattened steel pipes by competing Bauschinger effect and strain hardening during pipe-forming

**DOI:** 10.1038/s41598-019-50328-4

**Published:** 2019-09-30

**Authors:** Dae Woong Kim, Wan-Keun Kim, Jin-ho Bae, Won-Doo Choi, Hyoung Seop Kim, Sunghak Lee

**Affiliations:** 1 Center for Advanced Aerospace Materials Pohang, University of Science and Technology, Pohang, 790-784 Korea; 2 POSCO Computational Optimization of API Steels Project Team Technical Research Laboratories, POSCO, Kwangyang, 545-875 Korea; 3 Department of Advanced Materials Engineering, Hanbat National University, Daejeon, 305-719 Korea

**Keywords:** Mechanical properties, Metals and alloys

## Abstract

Since flattened steel sheets often show the unexpectedly lower or higher yield strength than leveled sheets, unceasing efforts have been made to accurately predict the yield strength in pipe-forming industries. In the present investigation, the yield strength of line-pipe or casing-pipe steels was predicted by competing Bauschinger effect and strain hardening occurred during the pipe-forming. Yield drop (YD) and yield rise (YR) parameters were newly defined from cyclic simulation analyses of outer and inner walls of pipes to express more reasonably the Bauschinger effect and strain hardening. The YD increased abruptly until the pre-strain of about 1%, and then saturated, while the YR increased linearly with increasing pre-strain. By combining the YD and YR, the variation in yield strength (Δσ) showed a down-and-up behavior as the Bauschinger effect and strain hardening were dominant at low and high pre-strains, respectively, and plausibly explained the relationship of Δσ and piping strain used in pipe-forming industries. According to the microstructural analyses related to the down-and-up Δσ behavior, the polygonal ferrite reduced the yield-strength reduction in the low pre-strain range, whereas the granular bainite or pearlite expanded it. This yield strength prediction coupled with microstructural analyses provide a good idea for designing and reliably predicting the yield strength of in various steel pipes.

## Introduction

Line-pipes used widely for the long-distance transportation of crude oil and natural gas have been produced by hot-rolling, coiling, leveling, and pipe-forming procedures, and their mechanical properties are evaluated after the flattening of a pipe wall segment. After the pipe-forming, pipes’ outer and inner layers in the walls experience repeatedly tensile and compressive strains^1–4^, respectively. Because of these different strain histories, the flattened segment of pipe walls often show the unexpectedly much lower or higher yield strength than that of the leveled sheets^5^. Since the pipe-forming, flattening, mechanical-property test procedures and resultant feedbacks usually consume a prolonged time and expensive costs, great efforts have been made for preventing or minimizing the large variation of yield strength in steel-making and pipe-forming industries.

The yield strength reduced under repeated strain histories is generally explained by a Bauschinger effect induced from back stresses due to the increment of mobile dislocations^6,7^. Line-pipe steels consist of polygonal or quasi-polygonal ferrite (PF or QPF) as a major microstructure in low-strength steels such as API X60-grade steels, and granular bainite (GB), acicular ferrite (AF), and bainitc ferrite (BF) become major microstructures as the strength grade increases. In ultra-high-strength API X100-grade steels or the higher ones, martensite-austenite constituent (MA) is also utilized^8–10^. These microstructures influence both Bauschinger effect and strain hardening, and their volume fractions appropriately control the yield-strength level. Thus, microstructural effects on yield strength should be carefully analyzed by a competing mechanism of Bauschinger effect and strain hardening. A pipe-forming strain expressed by a ratio of thickness/diameter (t/D) also influences this competition^11–13^. It has been well recognized that the yield strength steadily increases with increasing t/D^11,12^, but the unexpected reduction of yield strength is often found in very low t/D levels by the predominantly-working Bauschinger effect^13^. In order to define and clarify the competing mechanism, therefore, the investigation of microstructural effects on yield-strength variation is essentially needed, but only limited information on roles of microstructures and t/D is available until now.

In the present investigation, line-pipe or casing-pipe steel sheets having different constituent phases were made by varying steel compositions and coiling temperatures, and the Bauschinger effect and strain hardening were quantified by cyclic simulation tests of their leveled sheets to predict yield strength of the pipes without actually conducting the pipe-forming, flattening, and tensile testing. Repeated tensile and compressive strains undergone in outer and inner walls of the pipes were evaluated by newly-defined yield drop and rise parameters, respectively, which were combined to predict the yield-strength variation as a function of pre-strain, irrespective of pipe-forming processes. The resultant yield-strength variation data were then used for understanding effects of microstructural characteristics on Bauschinger effect and strain hardening. This study is expected to contribute to yield-strength designs of the line-pipe or casing-pipe steel sheets in relation to microstructural characteristics and to the reduction of unnecessary spending of time.

## Results

### Microstructures of API X65-grade line-pipe and API J55-grade casing-pipe steels

**Figure 1:**
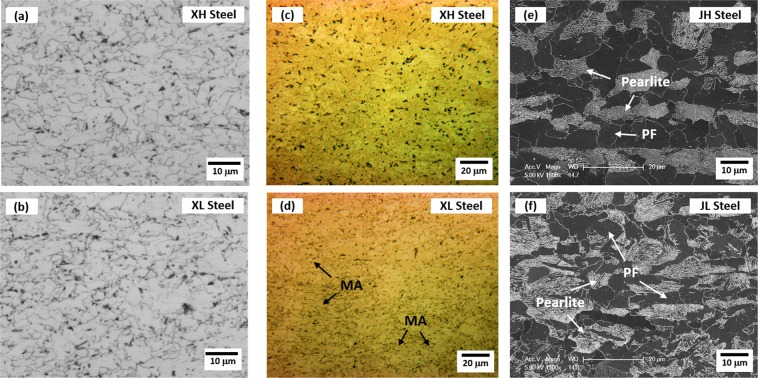
Optical micrographs (L-S plane) of the (**a,c**) XH and (**b,d**) XL steel specimens and SEM micrographs of the (**e**) JH and (**f**) JL specimens. The XH and XL specimens consist mainly of PF and GB, respectively, although the optical micrographs are not enough to clearly define microstructures. (**c**) and (**d**) show LePera-etched optical micrographs of the XH and XL specimens, respectively. A very few MAs are observed in the XL specimen as indicated by arrows in (**d**). Both JH and JL specimens consist of PF and pearlite without any bainitic microstructures.

Optical and SEM micrographs of the commercial API X65-grade line-pipe and API J55-grade casing-pipe steels are shown in Fig. 1(a–f). Here, the X65 steel sheets coiled at high and low temperatures are referred to as ‘XH’ and ‘XL’, respectively, and the J55 steel sheets coiled at high and low temperatures are referred to as ‘JH’ and ‘JL’, respectively. The XH and XL specimens consist mainly of PF and GB, respectively (Fig. 1(a,b)). MAs are not found in the LePera-etched optical micrograph of the XH specimen (Fig. 1(c)), while a very few MAs (volume fraction; less than 0.2%) are found in the XL specimen (arrow marks in Fig. 1(d)). According to SEM micrographs (Fig. 1(e,f)), both JH and JL specimens consist of PF and pearlite without any bainitic microstructures.

**Figure 2:**
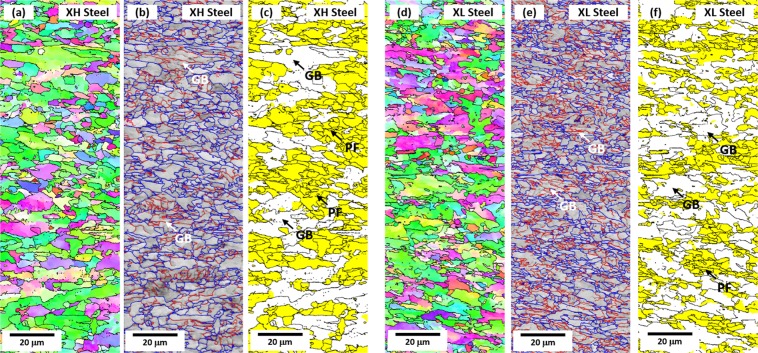
EBSD inverse pole figure (IPF), image quality (IQ), and grain orientation spread (GOS) maps of the (**a**–**c**) XH and (**d**–**f**) XL specimens. The PF area is defined in the GOS maps based on 3°-misorientation category as shown in yellow areas in (**c**) and (**f**), and its fractions are measured to be 62.8% and 54.8% in the XH and XL specimens, respectively. GB grains are coarser than PF grains.

The EBSD analysis was performed to define and quantify the XH and XL steel microstructures. Figure 2(a–f) shows inverse pole figure (IPF), image quality (IQ), and grain orientation spread (GOS) maps of the XH and XL specimens. Coarse microstructures containing substructures are observed together with fine randomly-oriented microstructures (Fig. 2(a,b,d,e)). According to the GOS maps (Fig. 2(c,f)), high-angle boundaries are distinguished by the misorientation of 15°, and the PF is regarded to be grains having misorientations of 3° or smaller^14^. Based on this classification by the GOS, the PF is distinguished from the GB, BF, and AF containing substructures. The PF is colored in yellow by the misorientation category of 3°, and its measured volume fraction is 62.8% and 54.8% in the XH and XL specimens, respectively. GB grains are coarser than PF grains.

Table 1 shows the quantitative data measured from Figs 1, 2. The XH specimen contains 62.8% of PF and 37.2% of GB. The XL specimen shows the lower PF fraction (45.0%) and higher GB fraction (54.8%) than the XH specimen. The grain size effect is not considered because the overall grain size differentiated by the misorientation of 15° is similar in the XH (11.0 μm) and XL (10.5 μm) specimens. The JH specimen composed of 63.2% of PF and 36.8% of pearlite shows the higher PF and lower pearlite fractions than the JL specimen. The effect of cementite lamellar spacing is not considered because it is similar in the JH (0.211 μm) and JL (0.215 μm) specimens. These microstructural differences enable the comparison of Bauschinger effect and strain hardening between the four steel specimens.

**Table 1:** Volume fractions of polygonal ferrite (PF), granular bainite (GB), pearlite, and martensite-austenite constituent (MA), and average grain size in the XH, XL, JH, and JL steel specimens. (unit: %).

Steel	PF	GB	Pearlite	MA	Grain Size (μm)
XH	62.8	37.2	—	—	11.0 ± 7.9
XL	45.0	54.8	—	0.2	10.5 ± 6.3
JH	63.2	—	36.8	—	9.71 ± 5.05
JL	52.8	—	47.2	—	9.28 ± 5.03

### Effects of PF and GB on Bauschinger effect and strain hardening in line-pipe steels

**Figure 3:**
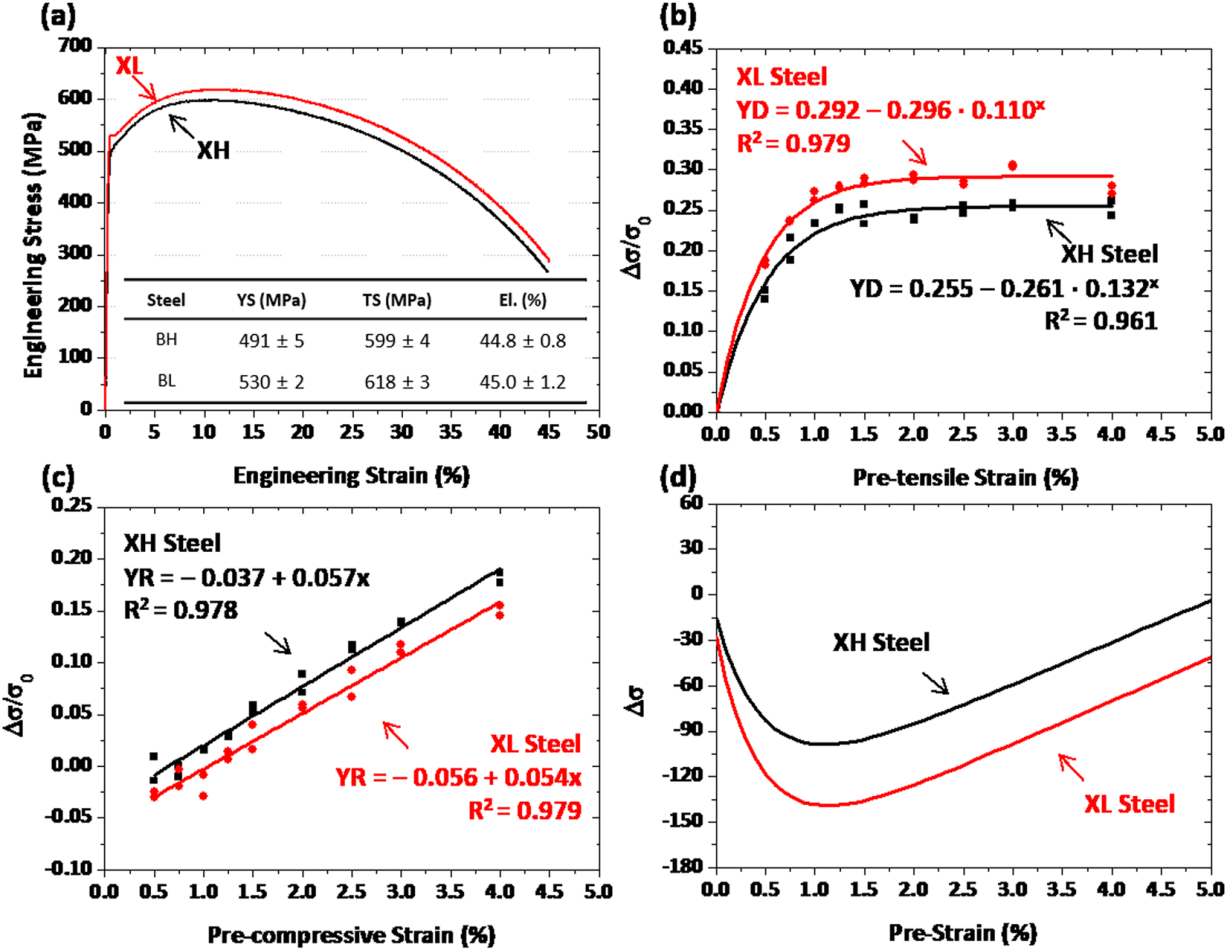
(**a**) Engineering tensile stress-strain curves, (**b,c**) yield drop (YD) and yield rise (YR) parameters measured at nine different pre-strains (0.5, 0.75, 1.0, 1.25, 1.5, 1.75, 2.0, 3.0, and 4.0%), and (**d**) change of yield strength (Δσ) measured by combining the YD and YR parameters as a function of pre-strain for the XH and XL specimens. The Δσ starts to decrease at low pre-strains, and then increases steadily after pre-strains of 1.0~1.5%. This is because the Bauschinger effect becomes saturated as the pre-strain increases, while the strain hardening increases linearly.

Room-temperature engineering tensile stress-strain curves of the XH and XL specimens are shown in Fig. 3(a), and the measured tensile properties are described inside the figure. The XH and XL specimens satisfies a minimum level of yield strength (448 MPa), and their curve shape is similar. Figure 3(b) shows plots of yield drop (YD) parameters as a function of pre-strain. The YD increases abruptly until the pre-strain of about 1.0%, and then saturates to a certain value. These YD curves are fitted into an equation of *y* = *a* − *b·c*^*x*^, where *a, b*, and *c* are constants related to the YD saturation. Coefficients of determination, R^2^, are higher than 0.96 in the XH and HL specimens, which indicates the excellent curve-fitting. The YD of the XL specimen whose GB fraction is higher than that of the XH specimen is higher throughout the whole pre-strain range. This implies the higher Bauschinger effect in the GB than in the PF.

Figure 3(c) shows plots of yield rise (YR) parameters measured at nine pre-strains. The YR increases linearly with increasing pre-strain, and can be fitted to a linear-type equation of *y* = *a* + *b·x*, where *a* and *b* are a constant and a slope related to the extent of strain hardening, respectively. The YR is higher in the XH specimen throughout the whole pre-strain range than in the XL specimen. This indicates that the increased amount of yield strength after the compression-tension-tension cyclic simulation test is larger in the XH specimen, whereas the strain hardening rate during the 4%-tensile deformation is similar in both XH and XL specimens (Fig. 3(a)). Based on the YR data (Fig. 3(c)), the YR values at the pre-strain of 4% are 0.337 and 0.237 in the XH and XL steels, respectively. When they are multiplied by the yield strength of each steel, the yield strength increases estimated after the compression-tension-tension cyclic simulation test are 94 MPa and 84 MPa in the XH and XL specimens, respectively.

The yield strength is generally influenced by microstructural parameters including grain sizes, volume fractions of precipitates, and dislocation densities^15–19^. Among them, the major factor affecting the yield-strength variation after the cyclic compression-tension test is the density of dislocations, in particular immobile dislocations which greatly affect the hardening^20^. The larger increase of yield strength in the XH specimen, whose PF fraction is higher than that of the XL specimen, indicates that the PF contributes more to the yield-strength increase than the GB. Since the PF formed by the diffusion at the higher temperature is softer than the GB^21^, the deformation is concentrated more to the PF by the strain partitioning^22^, and generates more immobile dislocations inside the PF, thereby leading to the larger yield-strength increase in the XH specimen.

Final mechanical properties of pipe-formed and flattened sheets can be obtained by combining overall properties of outer and inner walls. As aforementioned in the section 2.3, the Bauschinger effect and strain hardening are predominant in the outer and inner walls, respectively, and are expressed by the YD and YR parameters, respectively. Figure 3(d) shows the change of yield strength (Δσ) measured by combining the YD and YR parameters for the XH and XL specimens after the YD curves are turned upside down. The Δσ starts to decrease at low pre-strains, and then increases steadily after pre-strains of 1.0~1.5%. This is because the Bauschinger effect becomes saturated with increasing pre-strain, while the strain hardening increases linearly. This also indicates that the Bauschinger effect and strain hardening are predominant at low pre-strains and the higher pre-strains, respectively. The Δσ curve shape is similar in both XH and XL specimens, but the Δσ fluctuates more largely in the XL specimen. The yield strength of the XL specimen decreases more largely in the low pre-strain range by the larger Bauschinger effect together with the smaller strain hardening. Thus, the yield strength of the XL specimen can be largely reduced after the pipe-forming and flattening.

### Effects of PF and pearlite on Bauschinger effect and strain hardening in casting-pipe steels

**Figure 4:**
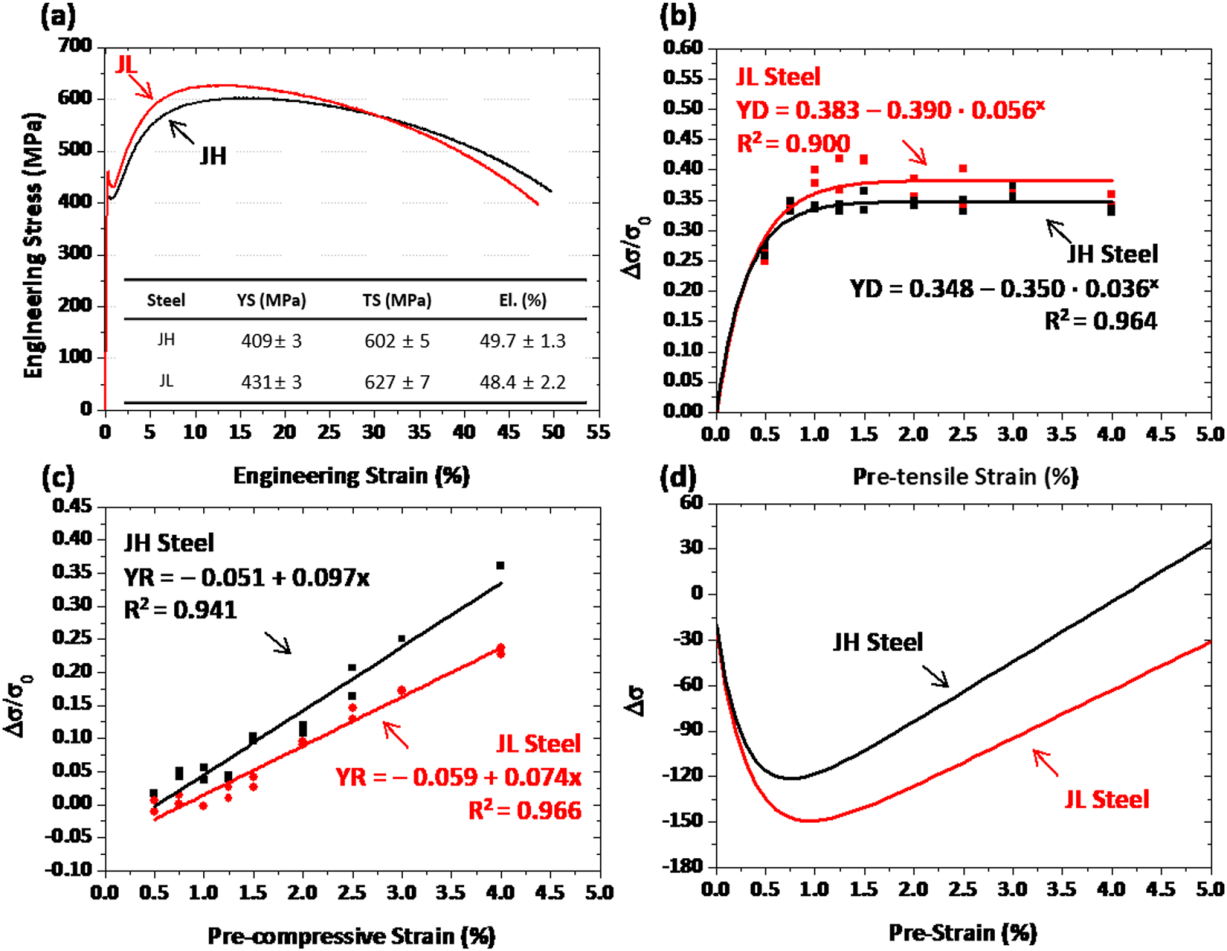
(**a**) Engineering tensile stress-strain curves, (**b,c**) yield drop (YD) and yield rise (YR) parameters measured at nine different pre-strains (0.5, 0.75, 1.0, 1.25, 1.5, 1.75, 2.0, 3.0, and 4.0%), and (**d**) change of yield strength (Δσ) measured by combining the YD and YR parameters as a function of pre-strain for the JH and JL specimens.

Tensile stress-strain curves of the JH and JL specimens are shown in Fig. 4(a). The yield strength of both JH and JL specimens satisfy the minimum yield-strength level (379 MPa), while the yield-point phenomenon appears in their curves. Overall properties are similar in both specimens. The YD and YR parameters were measured at nine different pre-strains, and are plotted in Fig. 4(b). In both specimens, the YD increases initially until it saturates to a certain value (Fig. 4(b)), like in the XH and XL specimens. The YR increases steadily in a linear mode with increasing pre-strain (Fig. 4(c)). These YD and YR curves are fitted to equations of *y* = *a – b·c*^*x*^ and *y* = *a* + *b·x*, respectively, while R2 values are very high over 0.95. The JH specimen whose PF fraction is higher than that of the JL specimen shows the lower YD and the higher YR throughout the whole pre-strain range. This implies that the Bauschinger effect is higher in the pearlite than in the PF because of dislocation pile-ups in the pearlite, while the strain hardening is higher in the PF because of the strain partitioning^22^. Figure 4(d) shows the Δσ measured by combining the YD and YR parameters. The Δσ curves of the JH and JL specimens show a down-and-up shape, like in the XH and XL specimens. The Δσ fluctuates more largely in the JL specimen as its yield-strength well is deeper, thereby resulting in the larger yield-strength reduction after the pipe-forming and flattening.

## Discussion

The Bauschinger effect and strain hardening induced from the subjected strain histories were evaluated by the YD and YR parameters, respectively, and the yield-strength variation of the pipes was estimated by combining these parameters, which were varied with pre-strains and steel microstructures.

### Competing mechanism of bauschinger effect and strain hardening

During the pipe-forming and flattening processes, the inner and outer walls of the pipe are deformed under tensile-compressive and compressive stress states, respectively, and each strain is dependent on the t/D^23^. The yield strength of the flattened sheet is determined by a competing mechanism of Bauschinger effect and strain hardening, which are reasonably expressed by the YD and YR parameters, respectively, in the present investigation. Interestingly, the YD increases at low pre-strains, and then saturates to a certain value at the higher pre-strains above 1.0~1.5% (Figs 3(b), 4(b)). This peculiar YD saturation can be interpreted by the movement of mobile dislocations^6^. Mobile dislocations can move reversibly during the strain-path change, whereas immobile dislocations are non-reversible and generating dislocation forests^20^. Only mobile dislocations have potentials to recombine positive- and negative-signed dislocations as they glide along reversible directions. In the initial deformation stage, mobile dislocations become populated abruptly^21^, although the overall density of dislocations rises as the accumulated strain increases. In the later deformation stage, the number of mobile dislocations becomes saturated while immobile dislocations start to form^20^, thereby resulting in the saturation of Bauschinger effect.

It is also noted that the YR increases steadily in a linear mode as the pre-strain increases (Figs 3(c), 4(c)). This linear-type YR increase is interpreted by the generation of immobile dislocations^20^. Different-signed dislocations gliding on the same slip plane can be annihilated by combining them as they move along reversed directions^24^. When dislocations gliding on different planes meet together, forest-type dislocations can be formed^20^. These dislocations are immobile, and acts as a strengthening mechanism to cause the increase of yield strength. As the compression-tensile deformation amount increases, the number of immobile dislocations increases nearly proportionally, which results in the linear-type YR increase, as shown in Figs 3(c), 4(c). However, this increasing trend of YR or yield strength is different from that of the strain hardening occurring in stress-strain curves. For example, the flow stress at the 4%-deformation without the pre-strain is higher in the JL specimens than in the JH specimen (559 MPa *vs*. 524 MPa (Fig. 4(a)), whereas the yield strength calculated from Fig. 4(c) at the pre-strain of 4% is higher in the JH specimens than in the JL specimen (533 MPa *vs*. 547 MPa). This result indicates that the strain hardening occurring in the repeated cyclic deformation is different from the strain hardening occurring in typical stress-strain curves, and that it might be mainly affected by the microstructures.

Since the yield strength of the pipes is estimated by combining the yield strengths of the outer and inner walls where the Bauschinger effect and strain hardening are respectively predominant, the yield-strength variation (Δσ) can be expressed by combining the YD and YR parameters, as shown in Figs 3(d), 4(d). The Δσ shows a down-and-up behavior as the Bauschinger effect and strain hardening are dominant at low and high pre-strains, respectively, because piled-up dislocations change with the pre-strain. At low pre-strains, the back stress becomes activated by forming many mobile dislocations, thereby resulting in the high YD and consequently in the decrease of yield strength. At high pre-strains, many mobile dislocations become immobile and tangled to increase their number. When the loading is reversed, the back stress does not work sufficiently, and the strain hardening starts to override the Bauschinger effect, thereby leading to the yield-strength increase. As the YR increases steadily with increasing pre-strain, the yield strength of the pipes becomes higher at a certain pre-strain level than that of the leveled sheets.

The flattened pipes show unexpectedly the lower or higher yield strength than the leveled sheets, and thus most of researchers working in the pipe-forming industries have made a great deal of effort to accurately predict the yield strength^25–27^. They recognize the dominance of Bauschinger effect and strain hardening at low and high levels of piping strain (t/D), respectively, based on which they conduct modeling approaches on yield-strength prediction^23^. However, an abrupt change in Bauschinger effect in the low t/D range cannot be sufficiently considered in the modeling studies, although it clearly appears in the pre-strain range of 1.0~1.5%, as shown in Figs 3(b), 4(b). As the t/D increases, the amount of deformation occurring in the outer and inner walls increases, which is consistent with the increasing tendency of pre-strain^11,12^.

**Figure 5:**
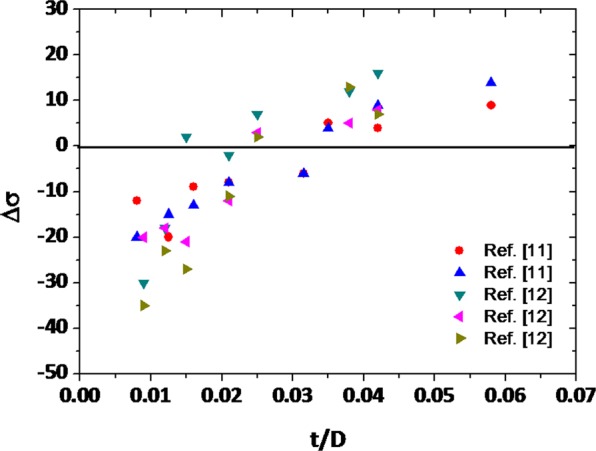
The yield-strength variation (Δσ) data collected from previous research papers^11,12^ as a function of *t/D*. The Δσ shows minus values in the low t/D range, and increases continuously with increasing *t/D* to become plus values at a certain t/D.

Various pipe-forming processes including Electric Resistance Welding (ERW), UOE, and spiral forming have been applied to line-pipe steels, and the yield-strength variation after the pipe forming varies slightly with the processes, although the overall trend is similar. The yield-strength variation (Δσ) data are collected from previous research papers^11,12^ on pipe-formed and flattened sheets having different t/D, and are shown as a function of t/D in Fig. 5. The Δσ shows minus values in the low t/D range, and increases continuously with increasing t/D to become plus values at a certain t/D. The modeling studies do not sufficiently interpret the Δσ data in the low t/D range which show relatively large deviations in Δσ, while they explain the overall increasing trend of Δσ with increasing t/D in the somewhat high t/D range. In fact, the down-and-up behavior of Δσ in the low t/D range is observed steadily in the enormous Δσ data of steel- or pipe-making industries, but its cause has hardly been explained yet.

In the present investigation, overall properties of pipes are predicted by combining properties of inner and outer walls of the pipe. Since commercial pipe forming processes applied to line-pipe steels are quite complicated, the prediction method of the Δσ of the pipes is suggested as a desirable and simple method by combining the YD and YR parameters as well as an impressive relationship between the Δσ and t/D (or pre-strain), which have not been reported in preceding researches on steel-pipe-forming. It reliably explains this Δσ behavior by the dominance of Bauschinger effect in the low t/D range. It is meaningful to understand effects of piping strains on yield-strength variation by combining the YD and YR parameters, which represent the Bauschinger effect and strain hardening, respectively. It is also useful to find which factor is more dominant in the variation of yield strength, and which steel is more suitable in the present prediction of the Δσ of the pipes.

### Effects of microstructure on Bauschinger effect and strain hardening

The down-and-up behavior of Δσ curve can be effectively used for verifying effects of constituent microstructures. When the Δσ curves of the XH and XL specimens are compared (Fig. 3(d)), the yield strength of the XL specimen, whose PF fraction is lower (the GB fraction is higher) than that of the XH specimen (Fig. 2(c,f)), decreases more largely in the low pre-strain range (Fig. 3(d)) by the larger Bauschinger effect (Fig. 3(b)) and the smaller strain hardening (Fig. 3(c)), although it keeps increasing after the pre-strain of 1.0%. The reduction of yield strength of the XH specimen is smaller than that of the XL specimen, and becomes to disappear at the pre-strain of 5.0%. Thus, the PF is favorable for the decrease of Bauschinger effect and the increase of strain hardening, which plays an important role in decreasing the reduction of yield strength in the low pre-strain range, while the GB affects to expand the reduction of yield strength.

**Figure 6:**
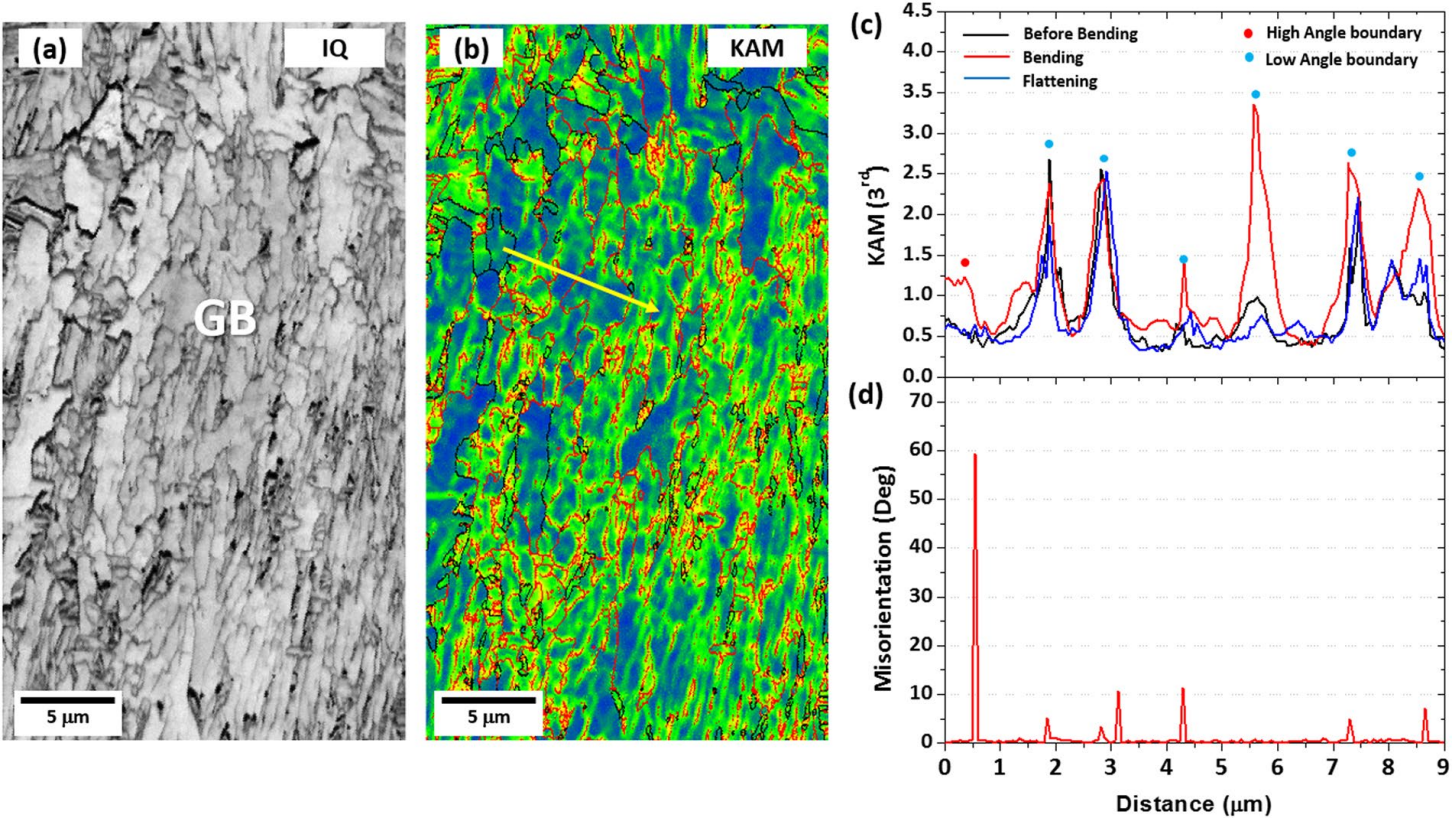
EBSD IQ and kernel average misorientation (KAM) maps of the GB area in the XL steel. KAM and misorientation were measured along yellow lines in (**b**) in the before-bending, bending (3%-tensile strain), and flattening (3%-compressive strain) stages, and the results are shown in (**c**) and (**d**), respectively. Overall KAM values are higher in the bending stage than in the before-bending or flattening stage. The higher KAM values in the bending stage appear at high- and low-angle boundaries whose misorientation line profiles are shown in (**d**).

In order to confirm the favorable effect of PF on strain hardening, the transmission electron microscopy (TEM) work can directly show the formation of dislocations and provide an evidence directly related to the Bauschinger effect such as dislocation pile-ups. However, it is hard to know whether dislocations form newly after the deformation or exist already before the deformation because it is almost impossible to show the dislocation behavior at the same observation areas before and after the tensile deformation. Thus, it is difficult to say whether dislocations induce a back stress or not. Thus, the three-point-bend test followed by EBSD kernel average misorientation (KAM) analysis was carried out on the GB area in the XL steel, and IQ and KAM maps are shown in Fig. 6(a,b). Detailed test and analysis methods are described in a previous study^28^. Line profiles of KAM value and misorientation obtained from a yellow-arrow line of Fig. 6(b) in stages of before-bending, bending (3%-tensile strain), and flattening (3%-compressive strain) are shown in Fig. 6(c,d). KAM values of the bending stage are higher than those of the before-bending stage or flattening stage (Fig. 6(c)). In the bending stage, the higher KAM values are found at high- and low-angle boundaries (Fig. 6(d)). This indicates that dislocations formed at low- and high-angle boundaries are mobile ones which can generate the back stress^28^. On the other hand, the PF deforms early in the low strain range, and then the yielding occurs, while most of dislocations are formed inside PF grains to induce a relatively low back stress^28^. Thus, dislocations formed inside PF grains lead to a weak Bauschinger effect, although the PF yields earlier by the strain partitioning than the GB.

**Figure 7:**
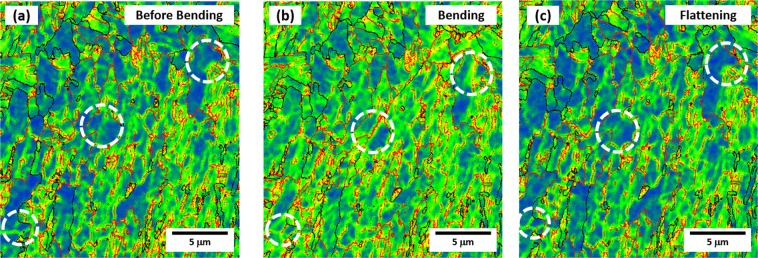
EBSD KAM maps of the GB area in the (**a**) before-bending, (**b**) bending, and (**c**) flattening stages of the XL steel. Low-angle boundaries, which are invisible in the before-bending stage as indicated by dashed circles in (**a**), appears newly in the bending stage, and then disappears in the flattening stage.

Figure 7(a–c) shows KAM maps of the GB area in the before-bending, bending, and flattening stages of the XL steel. It is interesting to note that low-angle boundaries, which are invisible in the before-bending stage as indicated by dashed circles in Fig. 7(a), appears newly in the bending stage (Fig. 7(b)), and then disappears in the flattening stage (Fig. 7(c)). It was reported that the back stress was typically generated by interactions between dislocations and obstacles such as grain boundaries^29^, while it was also induced by weakly-tangled dislocations^30^. The disappearance of low-angle boundaries in the flattening stage (Fig. 7(c)) is caused by the annihilation of weakly-tangled dislocations. In the GB area, thus, the Bauschinger effect increases by the back stress induced from weakly-tangled dislocations inside GB grains as well as dislocation pile-ups at low- or high-angle boundaries.

When the Δσ curves of the JH and JL specimens are compared (Fig. 4(d)), the yield strength of the JL specimen, whose PF fraction is lower (the pearlite fraction is higher) than that of the JH specimen (Fig. 1(e,f)), decreases more largely in the low pre-strain range (Fig. 4(d)) by the larger Bauschinger effect (Fig. 4(b)) and the smaller strain hardening (Fig. 4(c)), like in the XL specimen. This implies that the PF works for decreasing the reduction of yield strength in the low pre-strain range by decreasing the Bauschinger effect and by increasing the strain hardening. The pearlite expands the reduction of yield strength as its effect on Bauschinger effect is larger than that of the PF. This is because cementite lamellae existed in the pearlite work as pile-up sites for mobile dislocations to induce the back stress, as reported by many researchers^31,32^.

**Figure 8:**
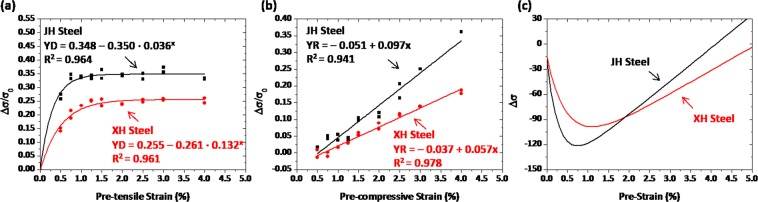
(**a,b**) yield drop (YD) and yield rise (YR) parameters and (**c**) change of yield strength (Δσ) measured by combining the YD and YR parameters as a function of pre-strain for the XH and JH specimens. In the low pre-strain range, the yield-strength reduction is deeper and more abrupt in the JH specimen than in the XH specimen. This implies that the pearlite contained in the JH specimen works more critically for the large yield-strength variation in the low pre-strain range than the GB contained in the XH specimen. As the pre-strain increases further, the Δσ increases more rapidly in the JH specimen because the pearlite shows the higher strain hardening than the GB, which implies the more favorable effects of pearlite on strain hardening.

When the curves of YD, YR, and Δσ curves of the XH and JH specimens, whose PF fraction is similar at about 63 vol.% while the another minor microstructure is GB or pearlite, respectively (Figs 1(e), 2(c)), are compared, both YD and YR are higher in the JH specimen than in the XH specimen (Fig. 8(a,b)). In the low pre-strain range, thus, the yield-strength reduction is deeper and more abrupt in the JH specimen than in the XH specimen (Fig. 8(c)). This implies that the pearlite contained in the JH specimen works more critically for the large yield-strength variation than the GB contained in the XH specimen. As the pre-strain increases further, the Δσ increases more rapidly in the JH specimen because the pearlite shows the higher strain hardening than the GB, which implies the more favorable effects of pearlite on strain hardening.

The above microstructural comparison results present a useful way to explain microstructural effects on yield-strength variation measured after the pipe-forming and flattening by competing the Bauschinger effect and strain hardening occurring in individual microstructures of PF, GB, and pearlite. According to the analyses of the down-and-up behavior of Δσ curves, the PF reduces the yield-strength reduction in the low pre-strain range, whereas the GB or pearlite expands it. These microstructural results provide a good idea for designing and reliably predicting the yield strength of in various steel pipes.

## Conclusions

In the present investigation, the yield strength of flattened line-pipe or casing-pipe steel sheets was predicted by competing Bauschinger effect and strain hardening quantified from cyclic simulation tests.

1. Yield drop (YD) and yield rise (YR) parameters were newly defined from cyclic simulation analyses of outer and inner walls of steel pipes to express more reasonably the Bauschinger effect and strain hardening occurred during the pipe-forming. The YD increased abruptly until the pre-strain of about 1%, and then saturated to a certain value, while the YR increased linearly with increasing pre-strain. YD and YR curves were well fitted into equations of y = a − b·c^x^ and y = a + b·x, respectively, where a, b, and c were constants.
2. Since the yield strength of the pipes was estimated by combining the yield strengths of the outer and inner walls where the Bauschinger effect and strain hardening were respectively predominant, its variation (Δσ) could be expressed by combining the YD and YR parameters. The Δσ showed a down-and-up behavior as the Bauschinger effect and strain hardening were dominant at low and high pre-strains, respectively, and plausibly explained the relationship of Δσ and piping strain generally used in steel-pipe-forming researches.
3. In X65-grade line-pipe steels composed of polygonal ferrite (PF) and granular bainite (GB), the GB was more favorable for the increase of Bauschinger effect and the decrease of strain hardening than the PF. This was because the back stress was increased in the GB by weakly-tangled dislocations as well as dislocation pile-ups at low- or high-angle boundaries. In J55-grade casing-pipe steels composed of PF and pearlite, the pearlite expanded the reduction of yield strength as its effect on Bauschinger effect was larger than that of the PF.
4. Since both YD and YR were higher in the J55 steels than in the X65 steels, the yield-strength reduction in the low pre-strain range was deeper and more abrupt in the J55 steels. This implied that the pearlite worked more critically for the large yield-strength variation than the GB. As the pre-strain increased further, the Δσ increased more rapidly in the J55 steels because the pearlite showed the higher strain hardening than the GB, which implied the more favorable effects of pearlite on strain hardening.

## Method

### Line-pipe or casing-pipe steel sheets

A commercial API X65-grade line-pipe steel (composition; (<0.05)C - (<1.5)Mn - (<0.6) (Ni + Cu) − 0.3Si - (<0.5) (Cr + Mo) − 0.03Al - (<0.15) (Ti + Nb + V) (wt.%)), minimum yield strength; 448 MPa, main microstructure; PF and GB) and an API J55-grade casing-pipe steel (composition; (<0.3)C - (<1.6)Mn - (<0.05) (Ti + Nb) (wt.%)), minimum yield strength; 379 MPa, main microstructure; PF and pearlite) were used in the present investigation. The steels were homogenized at 1215~1235 °C, and rolled at 1100~700 °C with a rolling reduction ratio of 80% or higher. After the finish rolling at 800~825 °C (in the austenite region above Ar_3_), they were cooled to 560~600 °C or 480~520 °C and coiled. The final thicknesses of the X65 and J55 steel sheets were 18 mm and 10 mm, respectively.

### Microstructural analyses and tensile tests

Steel microstructures of longitudinal-short-transverse (L-S) plane were examined by optical and scanning-electron microscopes after a 2%-nital etching. An etching in a LePera solution^33^ was also used to define martensite-austenite constituents (MAs). Electron back-scattered diffraction (EBSD) analysis (step size; 0.15 μm) was conducted on specimens electro-polished in a solution of 92%-CH_3_COOH + 8%-HClO_4_ at 32 V. Round tensile bars (gage diameter; 6.35 mm, gage length; 12.5 mm, transverse orientation) were obtained from the 1/2 thickness location of leveled sheets, and were tested at a strain rate of 5 × 10^−3^ s^−1^ at room temperature by using a universal testing machine (8801, Instron, USA, capacity; 100 kN).

### Cyclic simulation tests for estimating yield strength of the pipe

As the outer and inner walls were strained differently during pipe-forming and flattening procedures. A tensile-compression test, *i.e*., one-path change, was performed, to effectively show the Bauschinger effect caused by the back stress of dislocations. In addition, a compression-tension-tension test was also performed to show the strain hardening caused by the entanglement of dislocations. The strain (*ε*) varies with the outer diameter (*D*), thickness (*t*), and distance (*X*) from the sheet center as follow^34^:

1 $$\varepsilon (X)=2X/(D-t)$$

At a given *X*, *ε* increases as *D* decreases or *t* increases.

Cyclic simulation tests were performed to examine the Bauschinger effect and strain hardening. Nine pre-tensile and pre-compressive strains (*ε*_*pre*_) for the simulation tests of outer and inner walls, respectively, were determined as 0.5, 0.75, 1.0, 1.25, 1.5, 1.75, 2.0, 3.0, and 4.0% in consideration of strains varied with the sheet thickness location. Testing conditions and specimens were same to those of the tensile test.

**Figure 9:**
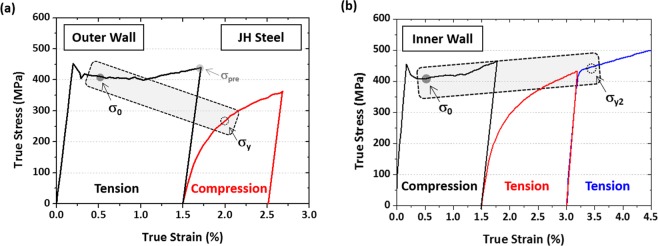
Typical (**a**) tension-compression and (**b**) compression-tension test curves to simulate the pipe-forming and flattening processes of the inner and outer walls, and definitions of tensile flow stress at the pre-strain of 1.5% (*σ*_*pre*_), compressive yield stress (σ_*y*_), initial tensile yield stress (*σ*_0_), and yield strength obtained after the compression-tension-tension cycle (σ_*y2*_). Yield drop parameter (YD) and yield rise parameter (YR) are quantified from tension-compression and compression-tension test curves, respectively.

The specimen was pre-strained under nine tensile strains to simulate the outer wall. In order to measure the yield strength during the compression, the minimum compressive strain was determined as 1%, and the yield strength was measured at the strain of 0.5% according to the API 5 L standards^35^. A Bauschinger stress parameter (BS) has been conventionally used for analyzing the Bauschinger effect varied with pre-strains^36^ as follow:

2 $$BS=({s}_{pre}-{s}_{y})/{s}_{pre}$$

where *σ*_*pre*_ and σ_*y*_ are the tensile flow stress at the pre-strain and the compressive yield stress, respectively. It often gives a different concept of yield-strength reduction as the strain-hardening effect is partly included in it^37^. In the present investigation, thus, a yield drop parameter (YD) is defined as follow^28^:

3 $$YD=({\sigma }_{0}-{\sigma }_{y})/{\sigma }_{0}$$

where *σ*_0_ is the pre-tensile yield strength. The 1.5% pre-tensile stain is exemplified in Fig. 9(a). Figures 3(b), 4(b) show the experimental test results at the pre-tensile strains from 0.5% to 4%. The YD can be more reasonably applied to the practical use of the pipe-forming than the BS because of the absence of the strain hardening in it.

The strain hardening occurred in the inner wall is expected to be same to that occurred in simple tensile flow curves which generally follows the Swift equation^38^. Since the inner wall experiences the compression-tension-tension cycle, however, its strain hardening is different from that of typical tensile flow curves as forest-type dislocations become populated during the compression-tension process. This increase of forest-type dislocations leads to the increase of strain hardening in the inner wall^39^. After the specimen was subjected to the compression-tension cycle under nine different strains to simulate the inner wall, it was tensioned to evaluate the yield strength. Here, a yield rise parameter (YR), which shows the strain hardening occurred during the pipe-forming, is newly defined by:

4 $$YR=({\sigma }_{y2}-{\sigma }_{0})/{\sigma }_{0}$$

where *σ*_0_ *and* σ_*y2*_ are the initial yield strength and the final yield strength obtained after the compression-tension-tension cycle, respectively. Figure 9(b) shows an example of true compression-tension-tension curves at the pre-strain of 1.5% in the JH steel specimen. Figures 3(c) and 4(c) show the experimental test results at the pre-compressive strains from 0.5% to 4%. This parameter expresses more reasonably the strain hardening occurred during the pipe-forming rather than that occurred in tensile flow curves.

## Data Availability

The data that support the findings of this study are available from the corresponding author upon reasonable request.
